# Body image disturbance, compensatory exercise, and exercise avoidance: cross-sectional conditional process associations in university students

**DOI:** 10.3389/fpsyg.2026.1917371

**Published:** 2026-08-31

**Authors:** Meng Zhang, Yujiao Wu, Tiansheng Xia

**Affiliations:** 1 School of Marxism, Henan Vocational College of Light Industry, Zhengzhou, China; 2 School of Art & Design, Guangdong University of Technology, Guangzhou, China

**Keywords:** body image disturbance, exercise behavior, exercise self-efficacy, goal-oriented motivation, shame

## Abstract

Body image disturbance is known to be associated with physical activity, yet the pathways linking them remain unclear. Drawing on self-regulation, self-conscious emotion and social cognitive theories, this study examines those pathways within a moderated dual-path model. In this cross-sectional study based on a convenience sample, data from 336 Chinese university students were collected via an online questionnaire and analyzed using confirmatory factor analysis (CFA; AMOS 24.0) for measurement validation and the PROCESS macro (Model 8; v4.1) for moderated mediation analysis with bootstrap confidence intervals (SPSS 27.0). The results showed that body image disturbance was positively associated with both compensatory exercise behavior and exercise avoidance behavior, reflecting a dual pattern of promotion and inhibition. Goal-oriented motivation partially accounted for the association between body image disturbance and compensatory exercise behavior, *b* = 0.184, 95% CI [0.119, 0.255], and shame partially accounted for the disturbance to avoidance association, *b* = 0.125, 95% CI [0.068, 0.191]. Exercise self-efficacy moderated both pathways. Compared with students reporting low self-efficacy, those reporting high self-efficacy showed a stronger association between body image disturbance and goal-oriented motivation and no significant association between body image disturbance and shame, and correspondingly stronger compensation and weaker avoidance. Because the data are cross-sectional, these findings describe conditional statistical associations rather than temporal or causal mechanisms.

## 1 Introduction

Against a backdrop of increasingly stringent aesthetic standards in contemporary society and the constant emphasis placed on them by the media, individuals' concern with their physical appearance has risen significantly (Sarda et al., 2025), and body image disturbance has become one of the key factors influencing the psychology and behavior of young people (López-Muñóz et al., 2025). This is particularly true among university

students, who are at a developmental stage characterized by intense self-identity formation and social comparison; their evaluation of body image is more susceptible to external standards and to the judgements of others (Guimond et al., 2026), thereby triggering persistent anxiety and dissatisfaction (Zhang et al., 2025). Such negative perceptions centered on one's physical appearance not only affect an individual's mental health but are also closely associated with their behavioral decision-making processes; this association is particularly complex in the context of sport, a setting closely associated with physical presentation (Burgon and Waller, 2024).

Existing research generally holds that body image disturbance does not simply inhibit or promote physical activity in a unidirectional manner (Monéger et al., 2022), but may instead produce a dual-path association through different psychological pathways (Burgon and Waller, 2024). On the one hand, according to self-regulation theory, when individuals perceive a gap between their actual self and their ideal self, they develop a motivation to bridge that gap (Macdonald and Palfai, 2008), thereby engaging in self-improvement through exercise and other means. On the other hand, based on self-conscious emotion theory, body image disturbance may also heighten an individual's sensitivity to others' evaluations, triggering negative emotions such as shame and leading to avoidance tendencies in exercise contexts involving bodily exposure or social comparison (Monéger et al., 2022; Mu and Berenbaum, 2019). Consequently, body image disturbance may simultaneously be associated with approach behaviors such as compensatory exercise and with avoidance behaviors such as exercise avoidance, forming a behavioral pattern characterized by internal tension.

On closer examination, the dual pathway described above may involve different types of psychological processes. On the one hand, goal-oriented motivation, as a positive motivation centered on self-improvement, helps individuals transform physical concerns into action goals (Box et al., 2019; Taylor et al., 2022), thereby promoting compensatory exercise behavior; on the other hand, shame, as a typical self-conscious emotion, undermines an individual's sense of self-worth and triggers avoidance tendencies (Kouri et al., 2023), thereby inhibiting exercise participation.

Furthermore, individual differences, particularly exercise self-efficacy, may play a key moderating role in this process (Ouyang et al., 2020; Wang et al., 2024). According to social cognitive theory, self-efficacy shapes how a situation is appraised, so students who differ in exercise self-efficacy may show different balances between the two pathways (An et al., 2024; Meixner et al., 2025; Niu et al., 2025). Therefore, incorporating exercise self-efficacy into the model helps to reveal the boundary conditions and the pathways through which body image disturbance is associated with exercise behavior.

The motivational and the emotional processes have each been examined in previous research, but they have rarely been brought together within a single model, and the moderating role of exercise self-efficacy across the two pathways has received little attention. The present study therefore integrates them empirically. Taking university students as its participants, and drawing on self-regulation theory, self-conscious emotion theory and social cognitive theory, it specifies a dual pathway model linking body image disturbance to exercise behavior, introduces goal-oriented motivation and shame as mediating variables, and further examines the moderating role of exercise self-efficacy. The aim is to describe the pathways along which body image disturbance is associated with compensatory and avoidance exercise behaviors, and thereby to provide a basis for further research on healthy exercise behavior.

## 2 Literature review and research hypotheses

### 2.1 Body image disturbance and exercise behavior

According to self-regulation theory, individuals constantly compare their actual self with their ideal self; when a gap exists between the two, this generates behavioral motivation to reduce that gap (Dalley et al., 2019). Body image disturbance is broader than body dissatisfaction, which refers only to a negative evaluation of appearance, and it entails a perceived discrepancy between the actual and the ideal body that intensifies the sense of self-inconsistency (Rodgers and DuBois, 2016). The body image disturbance questionnaire (Cash et al., 2004) measures body image disturbance in terms of appearance-related preoccupation and the accompanying distress and functional interference. It is usually accompanied by motivation to improve self-image through behavioral change. Exercise, as a controllable means of directly influencing body shape, often serves as a key statistical pathway for self-regulation (Shieh et al., 2015). Consequently, body image disturbance may promote increased engagement in exercise by stimulating compensatory motivation. Therefore, the following hypothesis is proposed:

- H1: Body image disturbance is significantly positively associated with compensatory exercise behavior.

At the same time, according to self-conscious emotion theory, body image disturbance increases an individual's self-focus in social situations (Castonguay et al., 2012), making them more susceptible to perceiving others' evaluations and experiencing negative emotional reactions. In exercise contexts, particularly those involving physical exposure or observation by others, individuals may experience discomfort due to concerns about being evaluated, leading them to avoid physical activity (More et al., 2019). Consequently, body image disturbance may induce avoidance behavior by increasing sensitivity to social evaluation. Therefore, the following hypothesis is proposed:

- H2: Body image disturbance is significantly positively associated with exercise avoidance behavior.

### 2.2 The mediating role of goal-oriented motivation

Achievement goal theory posits that an individual's behavior is significantly influenced by their goal orientation (Bardach et al., 2020), which primarily comprises mastery goals and performance goals (Ning, 2018). When individuals are oriented toward self-improvement and enhancing their abilities, they are more likely to view challenges as opportunities for growth and to achieve their goals through sustained effort (Varambally and Gangadhar, 2016). Body image disturbance may lead individuals to view improving their physique as a goal for self-improvement, thereby stimulating their goal-oriented motivation (Miller et al., 2022). Driven by this motivation, individuals are more likely to actively engage in exercise and achieve physical improvements through sustained behavior (De Meester et al., 2016). Therefore, the following hypothesis is proposed:

- H3: Goal-oriented motivation mediates the relationship between body image disturbance and compensatory exercise behavior.

### 2.3 The mediating role of shame

Shame is a typical self-conscious emotion that stems from an individual's negative evaluation of their overall self and is typically accompanied by self-denial and avoidance tendencies (Rockenberger and Brauchle, 2011). According to self-conscious emotion theory, when individuals are dissatisfied with their body image, they are more likely to experience shame in situations involving social comparison or being observed (Yuan et al., 2024). This emotion diminishes an individual's sense of self-worth and triggers avoidance behaviors to prevent further experiences of negative evaluation (Lucibello et al., 2025). In sporting contexts, particularly in activities requiring participation in public spaces or the exposure of the body, feelings of shame may significantly inhibit an individual's willingness to participate. Therefore, the following hypothesis is proposed:

- H4: Shame mediates the relationship between body image disturbance and exercise avoidance behavior.

### 2.4 The moderating role of exercise self-efficacy in the direct and indirect pathways

Social cognitive theory emphasizes that self-efficacy is a key factor influencing an individual's cognition, emotions and behavior (Charokopaki and Douros, 2025; Lucibello et al., 2025). Exercise self-efficacy reflects an individual's confidence in their ability to perform physical exercise tasks (Almutary and Tayyib, 2020; Neace et al., 2022). In the present study, exercise self-efficacy may moderate both the association between body image disturbance and shame and the association between body image disturbance and goal-oriented motivation, thereby producing a moderated mediation pattern.

Firstly, in the direct pathway, when individuals face body image disturbance, high exercise self-efficacy helps them take steps to reduce these concerns. Individuals with high self-efficacy are more likely to view the problem as controllable and improvable; they have greater confidence in their ability to complete exercise tasks, thereby enhancing compensatory exercise behaviors to address body image disturbance (Chu et al., 2021; Sheng et al., 2025); conversely, those with low self-efficacy may doubt the feasibility of change and fear negative evaluation while exercising, and so avoid it (Wang et al., 2024). Therefore, the following hypothesis is proposed:

- H5: Exercise self-efficacy positively moderates the association between body image disturbance and compensatory exercise behavior.
- H6: Exercise self-efficacy negatively moderates the association between body image disturbance and exercise avoidance behavior.

Secondly, according to social cognitive theory, individuals with high self-efficacy are more likely to transform appearance-related concerns of this kind into action-oriented cognitive strategies (Strachan and Brawley, 2008), rather than becoming bogged down in rumination about negative experiences. They are therefore more likely to read body image disturbance as a signal that change is needed and to form goal-oriented coping motivations such as improving posture or fitness (Honicke et al., 2020; Zhao et al., 2023). In contrast, individuals with low self-efficacy lack expectations of behavioral success; even when body image disturbance is present, they struggle to translate it into specific goals and action plans (Lynn et al., 2022), thereby weakening the mediating association. Based on this, the following hypothesis is proposed:

- H7: Exercise self-efficacy positively moderates the indirect association between body image disturbance and compensatory exercise behavior via goal-oriented motivation.

The experience of shame is typically accompanied by a sense of self-worth and anxiety regarding negative evaluations from others (Weingarden et al., 2017). Individuals with high self-efficacy possess a more robust sense of self-worth and confidence in their ability to regulate emotions, enabling them to cope more effectively with potentially threatening information (Kabak et al., 2026). Specifically, when confronted with body image disturbance, individuals with high self-efficacy are less prone to self-denigration or catastrophic thinking; consequently, the threshold for the activation of shame is higher and its intensity lower (Cumming et al., 2020; Huberty et al., 2008). Consequently, exercise self-efficacy may weaken the mediating role of shame between body image disturbance and exercise avoidance, thereby acting as a protective factor. Based on this, the following hypothesis is proposed:

- H8: Exercise self-efficacy negatively moderates the indirect association between body image disturbance and exercise avoidance behavior via shame.

**Figure 1:**
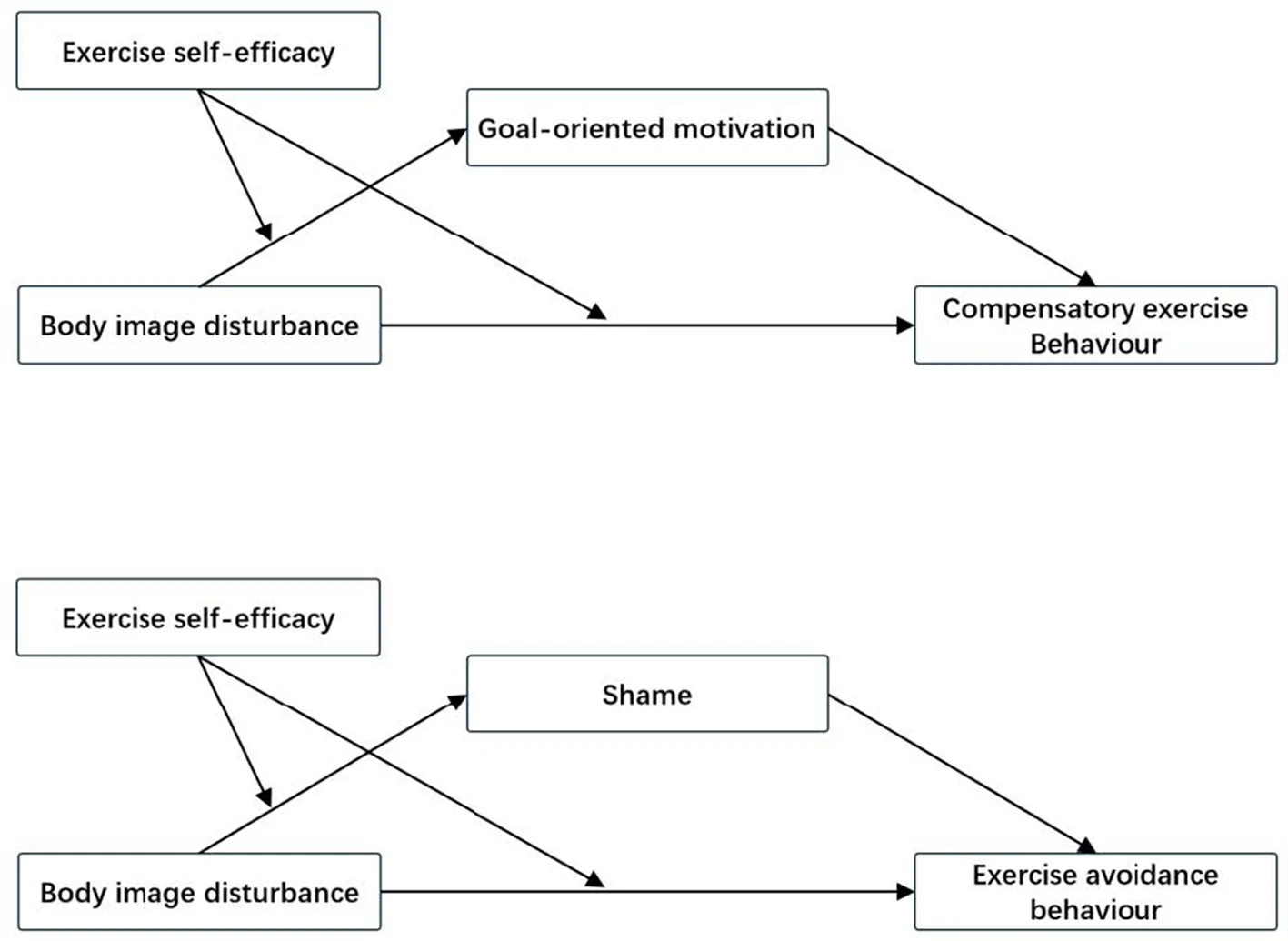
Research model.

A review of the literature reveals that existing research has examined individual exercise behavior from various perspectives, including body image, exercise motivation, self-consciousness and self-efficacy; however, most studies have focused on the direct association of a single variable with exercise behavior, and few have adopted an integrated approach to investigate how body image disturbance is simultaneously associated with both compensatory exercise behavior and exercise avoidance behavior through different psychological pathways. Furthermore, existing research lacks a unified, integrated analytical framework regarding the mediating pathways of goal-oriented motivation and shame, as well as the moderating role of exercise self-efficacy within these relationships. Therefore, it is necessary to build upon existing theories by introducing multi-path statistical pathways and moderating factors to construct a comprehensive model capable of explaining both the promoting pathway and the inhibiting pathway, thereby revealing more comprehensively the pathways through which body image disturbance is associated with exercise behavior. Accordingly, based on theoretical derivation and a review of the literature, this study has constructed the conceptual model shown in Figure 1 to systematically present the relationships between variables and their underlying pathways.

## 3 Method

### 3.1 Participants

Convenience sampling was employed, and 392 submitted questionnaires were obtained from current university students in China. They were from five geographic regions in China (South, North, East, Northeast, and Central China). Two data-quality rules, both specified before the data were examined, were then applied. First, a minimum completion time of 120 s was required. Under this rule, 39 questionnaires were excluded. Second, a single attention-check item was embedded among the measurement items, instructing respondents to select one designated response option; the 17 respondents who selected any other option were excluded. This left 336 questionnaires for analysis, a valid-completion rate of 85.71%. Of the analytic sample, 207 were female (61.61%) and 129 were male (38.39%). The study was approved by the Academic Ethics Review Committee of Guangdong University of Technology (protocol code GDUTXS20260147). Table 1 presents the demographic characteristics of the participants.

**Table 1:** Demographic characteristics (*n* = 336).

Characteristic	Items	Number	Percentage (%)
Gender	Male	129	38.39
	Female	207	61.61
Age	18–20 years	41	12.20
	21–22 years	92	27.38
	23–25 years	187	55.66
	26 years and older	16	4.76

The target sample size was determined on the basis of the most demanding test in the analytic plan: the interaction between body image disturbance and exercise self-efficacy. The sample exceeds the minimum of 200 cases recommended for confirmatory factor analysis (Kline, 2016). A sensitivity analysis for the achieved sample (*N* = 336) indicated that effect sizes of *f* ^2^ ≥ 0.024 could be detected with 80% power at α = 0.05, which remains within the small range. For the mediation component, the standardized *a* and *b* paths correspond to medium effects, for which bias-corrected bootstrap tests require approximately 71 cases (Fritz and MacKinnon, 2007).

The main survey was conducted between February and March 2026. The questionnaire was hosted on the WJX platform (https://www.wjx.cn/) and disseminated through the research team's WeChat contacts, group chats and Moments. Convenience sampling was used, and the eligibility criterion was current enrolment at a university in China. Participation was anonymous and voluntary, and all participants provided written informed consent before beginning the questionnaire. The platform was configured to accept only one submission per IP address.

### 3.2 Measures

The questionnaire was designed to measure the variables of the research model. It comprises two sections. The first covers demographic attributes, namely gender and age, and the second assesses the six variables included in the model and includes an attention-check item used to identify participants who did not answer attentively. All measures were adapted from established English-language instruments. None of the six is a previously validated Chinese-language instrument; all were translated and adapted for the present study, and permission was not required because no published scale is reproduced in full. Exercise self-efficacy, goal-oriented motivation and body image disturbance were adapted at the scale level from Chen et al. (2019) and Motl et al. (2000), the task-orientation subscale of Duda and Nicholls (1992) and Cash et al. (2004), respectively. For shame, compensatory exercise behavior and exercise avoidance behavior, items were selected from their source instruments (Hoppen et al., 2022; Knäuper et al., 2004; Yuan et al., 2024; Gámez et al., 2011) and reworded so that sport and exercise served as the explicit referent.

The scales were translated following a standard translation and back-translation procedure. Two bilingual postgraduate students majoring in psychology independently translated the original English items into Chinese, and a third bilingual researcher back-translated the preliminary Chinese version into English. The back-translated text was compared with the original items, and discrepancies in meaning and wording were resolved to produce the final Chinese version used in the main survey.

#### 3.2.1 Body image disturbance scale

Drawing on the Body Image Disturbance Questionnaire (Cash et al., 2004), the present study used six items to measure body image disturbance. Respondents were asked to rate the extent to which they were distressed by their body image, for example, “I frequently dwell on the flaws in my figure”. In this study, the Cronbach's α coefficient for the scale was 0.929.

#### 3.2.2 Exercise self-efficacy scale

The Exercise Self-Efficacy Scale, adapted from Chen et al. (2019) and Motl et al. (2000), was used. This scale measures respondents' confidence in their ability to engage in physical activity, as in the item “I can proactively schedule my workouts because I know how to do it”. In this study, the scale's Cronbach's α coefficient was 0.903.

#### 3.2.3 Shame

Four items were selected from the Guilt and Shame Questionnaire (GSQ-8; Hoppen et al., 2022). Participants rated their feelings of shame in exercise contexts, as in the item “I worry that others will look down on or despise me because of my athletic ability or physical performance”. In this study, the scale's Cronbach's α coefficient was 0.810.

#### 3.2.4 Goal-oriented motivation scale

Drawing on the task-orientation subscale of the Task and Ego Orientation in Sport Questionnaire (Duda and Nicholls, 1992), respondents were asked to assess their own exercise motivation, as in the item “I feel successful when I learn new skills in sports”. The scale comprises five items, each scored on a five-point Likert scale. In this study, the scale's Cronbach's α coefficient was 0.904.

#### 3.2.5 Compensatory exercise behavior

Five items were selected from the Compensatory Health Beliefs Scale (Knäuper et al., (2004) and from the compensatory-behavior content cataloged by Yuan et al. 2024). The items measure respondents' tendency to use exercise to compensate for eating or for missed activity, as in the item “When I eat more, I compensate by exercising extra.” In this study, the scale's Cronbach's α coefficient was 0.937.

#### 3.2.6 Exercise avoidance behavior

Five items were selected from the behavioral-avoidance content of the Multidimensional Experiential Avoidance Questionnaire (Gámez et al., 2011). Participants assessed their own exercise avoidance behaviors, as in the item “I try to avoid exercising when it makes me feel embarrassed or uncomfortable.” In this study, the scale's Cronbach's α coefficient was 0.929.

All items were answered on a five-point Likert scale from 1 (strongly disagree) to 5 (strongly agree). Each construct score was computed as the unweighted mean of its items, giving a possible range of 1 to 5, with higher scores indicating a higher level of the construct concerned.

### 3.3 Statistical analysis

Analyses used SPSS 27.0, AMOS 24.0 and PROCESS 4.1 (Hayes, 2022), and the analytic variables were observed composite scores (unweighted item means). Confirmatory factor analysis of the 33 items used maximum-likelihood estimation, the five ordered categories being treated as approximately continuous since all were used and non-normality was modest (item-level skewness −0.328 to 0.140; excess kurtosis −1.131 to −0.294). In the six-factor model, each item loaded only on its designated factor, with all cross-loadings and correlated residuals fixed to zero. As a sensitivity check, all models were re-estimated after removing three body image disturbance items covering avoidance or functional interference and one compensatory exercise item covering weight and appearance.

Model 4 was used for the two simple mediation analyses and Model 8 for the conditional process analyses; because PROCESS takes only one outcome at a time, the two pathways were estimated separately rather than as one combined model. Predictor and moderator were mean-centered before forming the interaction terms, and conditional associations were probed at the 16th, 50th and 84th percentiles of exercise self-efficacy, the PROCESS default (2.375, 3.250 and 4.000). Coefficients are unstandardized (b); the indirect associations and the index of moderated mediation use percentile bootstrap intervals from 5,000 resamples with a fixed seed.

The four equations were tested for homoscedasticity using the Koenker studentized Breusch-Pagan test (Breusch and Pagan, 1979) and White's test (White, 1980) tests, both indicated departures, so all were re-estimated with HC3 standard errors (Hayes and Cai, 2007; Long and Ervin, 2000) alongside ordinary least squares values. Influential cases were examined with Cook's distance, leverage and studentized residuals, and both indices of moderated mediation were re-estimated 336 times, omitting one case in turn.

## 4 Results

### 4.1 Assessment of potential common-method variance

Procedural remedies were adopted during questionnaire design and administration: participation was anonymous, and items belonging to different constructs were presented in separate modules. As a preliminary descriptive diagnostic, Harman's single-factor test was conducted. An unrotated exploratory factor analysis of the 33 measurement items returned six factors with eigenvalues greater than one, together accounting for 71.33% of the total variance. The first factor accounted for 25.14%, below the commonly used 40% threshold (Zhou and Long, 2004), indicating that the covariance among the items was not dominated by a single general factor. In addition, a sensitivity check was conducted by adding an unmeasured latent method factor with freely estimated loadings on all 33 items and constraining it to be orthogonal to all substantive latent factors (Podsakoff et al., 2003). The augmented model yielded χ^2^ = 609.62, df = 447, CFI = 0.979, TLI = 0.975, RMSEA = 0.033, and SRMR = 0.037. However, the solution converged at a parameter boundary, with the residual variance of one item approaching zero. Collectively, these results demonstrate that common method bias is not a severe confounding issue in the present dataset.

### 4.2 Measurement-model assessment and construct-validity evidence

The six-factor measurement model fitted the data well, χ^2^ = 691.87, df = 480, *p* < 0.001, χ^2^*/*df = 1.441, CFI = 0.972, TLI = 0.969, RMSEA = 0.036 with a 90% confidence interval of [0.030, 0.042], and SRMR = 0.038. Cronbach's alpha exceeded 0.70 for every latent variable, all standardized factor loadings exceeded 0.60, composite reliability exceeded 0.70 and average variance extracted exceeded 0.50 (Anderson and Gerbing, 1988; Fornell and Larcker, 1981), indicating adequate reliability and convergent validity. Table 2 reports the details.

**Table 2:** Reliability and convergent validity indicators (*n* = 336).

Construct	Item	Factor loading	Cronbach's alpha	AVE	CR
Exercise self-efficacy	ESE1	0.799	0.903	0.542	0.904
	ESE2	0.736			
	ESE3	0.730			
	ESE4	0.748			
	ESE5	0.684			
	ESE6	0.744			
	ESE7	0.753			
	ESE8	0.686			
Body image disturbance	BID1	0.783	0.929	0.694	0.931
	BID2	0.783			
	BID3	0.866			
	BID4	0.873			
	BID5	0.843			
	BID6	0.847			
Goal-oriented motivation	GM1	0.797	0.904	0.658	0.905
	GM2	0.770			
	GM3	0.753			
	GM4	0.874			
	GM5	0.854			
Shame	Shame1	0.693	0.810	0.520	0.812
	Shame2	0.667			
	Shame3	0.709			
	Shame4	0.806			
Compensatory exercise behavior	CEB1	0.842	0.937	0.749	0.937
	CEB2	0.848			
	CEB3	0.924			
	CEB4	0.934			
	CEB5	0.768			
Exercise avoidance behavior	EAB1	0.788	0.929	0.739	0.934
	EAB2	0.881			
	EAB3	0.816			
	EAB4	0.917			
	EAB5	0.888			

ESE, exercise self-efficacy; BID, body image disturbance; GM, goal-oriented motivation; CEB, compensatory exercise behavior; EAB, exercise avoidance behavior.

Comparisons with competing measurement models provided further evidence that the six constructs were empirically distinguishable. The hypothesized six-factor model fitted substantially better than the one-factor model, χ^2^ = 5795.00, df = 495, CFI = 0.300, TLI = 0.253, RMSEA = 0.179, and SRMR = 0.190. Fit was also poorer when body image disturbance was combined with exercise avoidance behavior, χ^2^ = 1992.17, df = 485, CFI = 0.801, TLI = 0.783, RMSEA = 0.096, and SRMR = 0.102, or with compensatory exercise behavior, χ^2^ = 2054.11, df = 485, CFI = 0.793, TLI = 0.774, RMSEA = 0.098, and SRMR = 0.103. The model combining goal-oriented motivation with compensatory exercise behavior yielded χ^2^ = 1453.53, df = 485, CFI = 0.872, TLI = 0.861, RMSEA = 0.077, and SRMR = 0.073, whereas the model combining shame with exercise avoidance behavior yielded χ^2^ = 966.25, df = 485, CFI = 0.936, TLI = 0.931, RMSEA = 0.054, and SRMR = 0.052.

Discriminant validity was supported. As shown in Table 3, every inter-factor correlation was smaller than the corresponding square root of the AVE (Fornell and Larcker, 1981), the highest correlation being 0.565 between shame and exercise avoidance behavior. All HTMT ratios were below 0.85, with the upper bound of the 95% bootstrap confidence interval also below 0.85 in every case, the highest being 0.676; HTMT values recalculated from polychoric correlations ranged from 0.071 to 0.576 (Table 4).

**Table 3:** Discriminant validity of the measurement model (*n* = 336).

Variables	ESE	BID	GM	Shame	CEB	EAB
ESE	0.736					
BID	−0.066	0.833				
GM	0.050	0.396	0.811			
Shame	−0.098	0.294	0.114	0.721		
CEB	0.066	0.336	0.510	0.120	0.865	
EAB	−0.095	0.339	0.187	0.565	0.204	0.860

**Table 4:** Heterotrait-monotrait (HTMT) ratio matrix (*n* = 336).

Variables	ESE	BID	GM	CEB	Shame	EAB
ESE	—					
BID	0.073	—				
GM	0.078	0.398	—			
CEB	0.071	0.344	0.539	—		
Shame	0.116	0.295	0.134	0.154	—	
EAB	0.108	0.346	0.193	0.203	0.576	—

ESE, exercise self-efficacy; BID, body image disturbance; GM, goal-oriented motivation; CEB, compensatory exercise behavior; EAB, exercise avoidance behavior. HTMT values below 0.85 indicate acceptable discriminant validity.

This item-reduction sensitivity test was conducted to verify the robustness of the empirical findings against arbitrary item selection bias. The reduced six-factor measurement model fitted the data well, χ^2^ = 479.35, df = 362, CFI = 0.980, TLI = 0.978, RMSEA = 0.031, SRMR = 0.038. Using the reduced composite scores, the indirect association of body image disturbance with compensatory exercise behavior through goal-oriented motivation was *b* = 0.176, 95% bootstrap CI [0.115, 0.242], and the indirect association with exercise avoidance behavior through shame was *b* = 0.119, 95% bootstrap CI [0.066, 0.179]. The two indices of moderated mediation were 0.150, 95% bootstrap CI [0.083, 0.223], and −0.072, 95% bootstrap CI [−0.127, −0.025]. All estimates fell within the confidence intervals of the corresponding primary analyses.

### 4.3 Descriptive statistics and correlations

Table 5 reports the descriptive statistics and the bivariate correlations among the six focal variables. Body image disturbance was positively correlated with goal-oriented motivation, shame, compensatory exercise behavior and exercise avoidance behavior, whereas exercise self-efficacy showed only weak correlations with the remaining variables.

**Table 5:** Descriptive statistics and correlations among the focal variables (*n* = 336).

Variable	M	SD	Skew	Kurt	1	2	3	4	5	6
1. BID	3.34	0.88	−0.15	−0.56	—					
2. ESE	3.21	0.75	−0.25	−0.42	−0.06	—				
3. GM	3.14	0.98	0.05	−0.80	0.37	0.05	—			
4. Shame	3.22	0.96	0.00	−0.62	0.26	−0.09	0.11	—		
5. CEB	3.34	1.01	−0.18	−0.73	0.32	0.05	0.50	0.13	—	
6. EAB	3.32	0.96	−0.14	−0.73	0.32	−0.09	0.18	0.50	0.19	—

BID, body image disturbance; ESE, exercise self-efficacy; GM, goal-oriented motivation; CEB, compensatory exercise behavior; EAB, exercise avoidance behavior. Skew, skewness, Kurt, excess kurtosis. All variables have a possible range of 1 to 5, and no variable had missing values. Correlations with an absolute value of 0.11 or above are significant at *p* < 0.05.

### 4.4 Analysis of mediating associations

#### 4.4.1 The mediating role of goal-oriented motivation in the association between body image disturbance and compensatory exercise behavior

In the regression analysis, body image disturbance was significantly and positively associated with goal-oriented motivation, *b* = 0.407, SE = 0.057, *t* = 7.168, *p* < 0.001, 95% CI [0.295, 0.519]. When body image disturbance and goal-oriented motivation were entered simultaneously, goal-oriented motivation was significantly and positively associated with compensatory exercise behavior, *b* = 0.451, SE = 0.052, *t* = 8.672, *p* < 0.001, 95% CI [0.349, 0.554], and the direct association between body image disturbance and compensatory exercise behavior remained significant, *b* = 0.186, SE = 0.058, *t* = 3.201, *p* = 0.002, 95% CI [0.072, 0.300], which indicated a partial mediating association. The bootstrap test further indicated that the indirect association of body image disturbance with compensatory exercise behavior through goal-oriented motivation was 0.184, 95% bootstrap CI [0.119, 0.255]. The model accounted for 13.3% of the variance in goal-oriented motivation and 26.8% of the variance in compensatory exercise behavior. Adjustment for gender and age category left the estimate essentially unchanged, *b* = 0.181, 95% bootstrap CI [0.117, 0.252].

#### 4.4.2 The mediating role of shame in the association between body image disturbance and exercise avoidance behavior

In the first-stage regression analysis, body image disturbance was significantly and positively associated with shame, *b* = 0.279, SE = 0.058, *t* = 4.831, *p* < 0.001, 95% CI [0.166, 0.393]. In the second-stage regression analysis, with body image disturbance and shame entered simultaneously, shame was significantly and positively associated with exercise avoidance behavior, *b* = 0.448, SE = 0.048, *t* = 9.413, *p* < 0.001, 95% CI [0.355, 0.542], and the direct association between body image disturbance and exercise avoidance behavior remained significant, *b* = 0.226, SE = 0.052, *t* = 4.342, *p* < 0.001, 95% CI [0.124, 0.329], which indicates a partial mediating association. The bootstrap test further indicates that the indirect association of body image disturbance with exercise avoidance behavior through shame was 0.125, 95% bootstrap CI [0.068, 0.191]. The model accounted for 6.5% of the variance in shame and 29.2% of the variance in exercise avoidance behavior. Adjustment for gender and age category again left the estimate unchanged, *b* = 0.125, 95% bootstrap CI [0.067, 0.191]. Relative to the total associations reported in Table 6, the indirect paths accounted for approximately half (0.184 of 0.369) and approximately a third (0.125 of 0.351), indicating associations of modest practical magnitude.

**Table 6:** Mediation analyses for the two pathways (*n* = 336).

Path	*b*	SE	*t*	*p*	95% CI
Model 1 (compensatory exercise behavior)					
BID → GM	0.407	0.057	7.168	< 0.001	[0.295, 0.519]
GM → CEB	0.451	0.052	8.672	< 0.001	[0.349, 0.554]
BID → CEB (total)	0.369	0.060	6.187	< 0.001	[0.252, 0.487]
BID → CEB (direct)	0.186	0.058	3.201	0.002	[0.072, 0.300]
BID → GM → CEB (indirect)	0.184	—	—	—	[0.119, 0.255]
Model 2 (exercise avoidance behavior)					
BID → Shame	0.279	0.058	4.831	< 0.001	[0.166, 0.393]
Shame → EAB	0.448	0.048	9.413	< 0.001	[0.355, 0.542]
BID → EAB (total)	0.351	0.057	6.212	< 0.001	[0.240, 0.463]
BID → EAB (direct)	0.226	0.052	4.342	< 0.001	[0.124, 0.329]
BID → Shame → EAB (indirect)	0.125	—	—	—	[0.068, 0.191]

BID, body image disturbance; GM, goal-oriented motivation; CEB, compensatory exercise behavior; EAB, exercise avoidance behavior. Coefficients are unstandardized (*b*). Confidence intervals for the regression paths are ordinary least squares intervals, and those for the indirect associations are percentile bootstrap intervals based on 5,000 resamples. *R*^2^ = 0.133 for goal-oriented motivation, 0.268 for compensatory exercise behavior, 0.065 for shame and 0.292 for exercise avoidance behavior.

Taking the above mediation results together, body image disturbance shows significant direct associations with the two exercise behaviors and significant indirect associations through goal-oriented motivation and shame, respectively. Hypotheses H1–H4 are therefore supported.

### 4.5 Analysis of moderating associations

#### 4.5.1 The moderating role of exercise self-efficacy in the association between body image disturbance and compensatory exercise behavior

**Figure 2:**
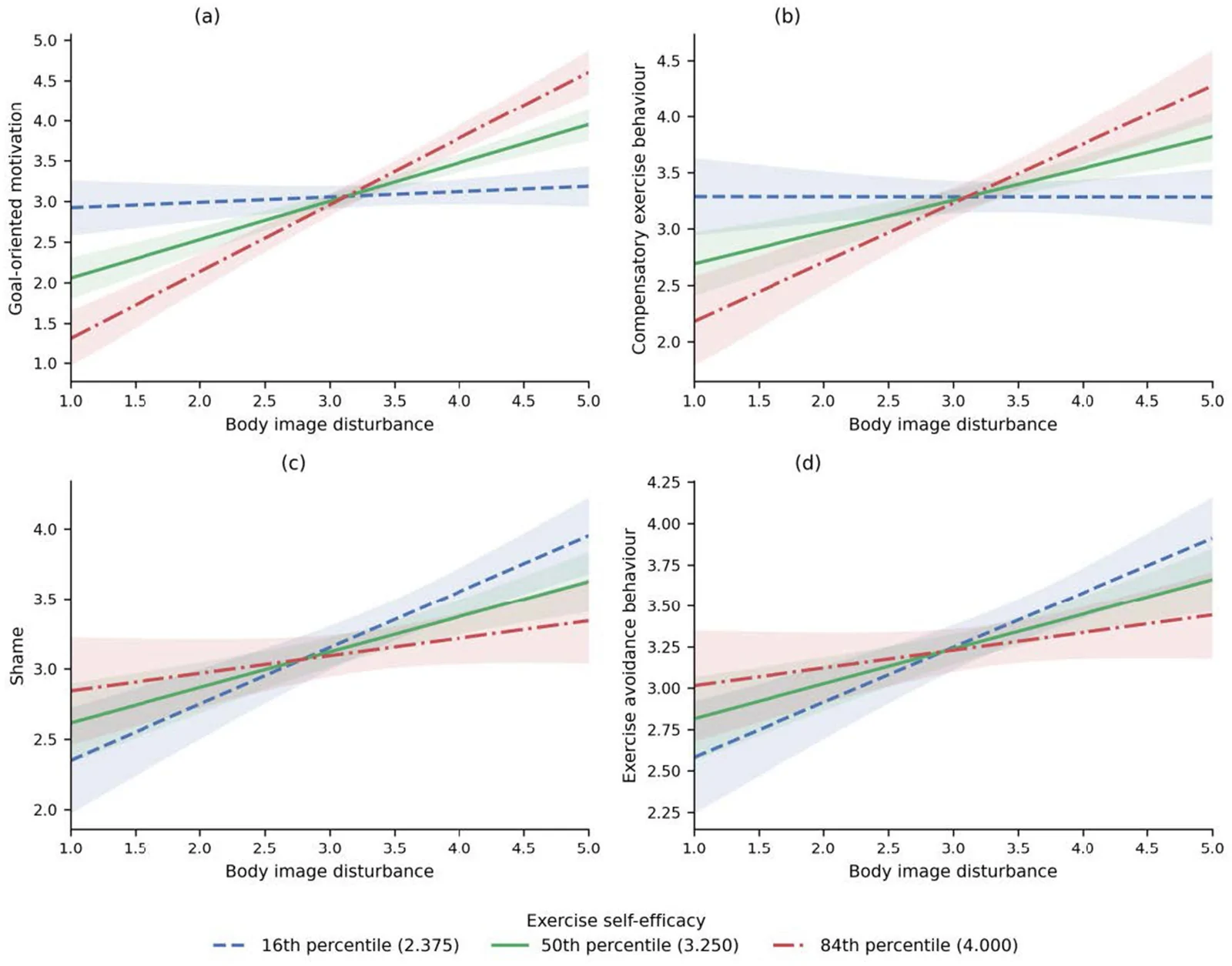
Simple slopes for the association of body image disturbance with **(a)** goal-oriented motivation, **(b)** compensatory exercise behavior, **(c)** shame and **(d)** exercise avoidance behavior, at the 16th (2.375), 50th (3.250) and 84th (4.000) percentiles of exercise self-efficacy. Shaded bands are 95% confidence intervals. Coefficients are unstandardized (*b*), *n* = 336.

In the model linking body image disturbance to compensatory exercise behavior through goal-oriented motivation, the interaction between body image disturbance and exercise self-efficacy was significantly and positively associated with goal-oriented motivation, *b* = 0.465, SE = 0.056, *t* = 8.299, *p* < 0.001, Δ*R*^2^ = 0.148, and with compensatory exercise behavior, *b* = 0.323, SE = 0.062, *t* = 5.221, *p* < 0.001, Δ*R*^2^ = 0.056. As shown in Table 7 and plotted in Figures 2a, b, the conditional associations were not significant at the 16th percentile of exercise self-efficacy and were significant and progressively stronger at the 50th and 84th percentiles; the conditional indirect association followed the same pattern. The index of moderated mediation was 0.153, 95% bootstrap CI [0.083, 0.225]. Exercise self-efficacy therefore positively moderates the indirect association of body image disturbance with compensatory exercise behavior through goal-oriented motivation.

**Table 7:** Conditional and indirect associations at three levels of exercise self-efficacy (*n* = 336).

Association	Level of ESE	*b*	95% CI	*p*
Model 1				
BID → GM	16th (2.375)	0.066	[−0.065, 0.197]	0.322
	50th (3.250)	0.473	[0.370, 0.576]	< 0.001
	84th (4.000)	0.821	[0.680, 0.962]	< 0.001
BID → CEB (conditional direct)	16th (2.375)	−0.001	[−0.133, 0.131]	0.988
	50th (3.250)	0.282	[0.166, 0.397]	< 0.001
	84th (4.000)	0.524	[0.357, 0.692]	< 0.001
BID → GM → CEB (conditional indirect)	16th (2.375)	0.022	[−0.018, 0.072]	—
	50th (3.250)	0.155	[0.086, 0.224]	—
	84th (4.000)	0.270	[0.153, 0.384]	—
Index of moderated mediation		0.153	[0.083, 0.225]	—
Model 2				
BID → Shame	16th (2.375)	0.401	[0.256, 0.546]	< 0.001
	50th (3.250)	0.252	[0.138, 0.366]	< 0.001
	84th (4.000)	0.125	[−0.031, 0.281]	0.116
BID → EAB (conditional direct)	16th (2.375)	0.332	[0.200, 0.464]	< 0.001
	50th (3.250)	0.211	[0.109, 0.313]	< 0.001
	84th (4.000)	0.107	[−0.029, 0.244]	0.124
BID → Shame → EAB (conditional indirect)	16th (2.375)	0.171	[0.107, 0.249]	—
	50th (3.250)	0.108	[0.052, 0.172]	—
	84th (4.000)	0.053	[−0.024, 0.133]	—
Index of moderated mediation		−0.073	[−0.131, −0.023]	—

BID, body image disturbance; ESE, exercise self-efficacy; GM, goal-oriented motivation; CEB, compensatory exercise behavior; EAB, exercise avoidance behavior. Coefficients are unstandardized (b). Conditional associations are probed at the 16th, 50th and 84th percentiles of exercise self-efficacy, which correspond to values of 2.375, 3.250 and 4.000. Confidence intervals for the conditional associations are ordinary least squares intervals, and those for the conditional indirect associations and for the index of moderated mediation are percentile bootstrap intervals based on 5,000 resamples.

#### 4.5.2 The moderating role of exercise self-efficacy in the association between body image disturbance and exercise avoidance behavior

In the model linking body image disturbance to exercise avoidance behavior through shame, the interaction was significantly and negatively associated with shame, *b* = −0.170, SE = 0.062, *t* = −2.740, *p* = 0.007, Δ*R*^2^ = 0.021, and with exercise avoidance behavior, *b* = −0.138, SE = 0.055, *t* = −2.530, *p* = 0.012, Δ*R*^2^ = 0.013. As shown in Table 7 and plotted in Figures 2c, d, the conditional associations were significant at the 16th percentile of exercise self-efficacy, attenuated at the 50th and no longer significant at the 84th; the conditional indirect association followed the same pattern. The index of moderated mediation was −0.073, 95% bootstrap CI [−0.131, −0.023]. Exercise self-efficacy therefore negatively moderates the indirect association of body image disturbance with exercise avoidance behavior through shame.

Both indices of moderated mediation were significant, so H5 to H8 were supported. With gender and age category included in every equation, the pattern of conditional associations was unchanged, and the indices of moderated mediation were 0.151, 95% bootstrap CI [0.081, 0.223], for the pathway through goal-oriented motivation and −0.074, 95% bootstrap CI [−0.132, −0.023], for the pathway through shame. Table 8 reports the regression coefficients for the two models, and Table 7 reports the conditional and indirect associations.

**Table 8:** Regression coefficients for the two moderated mediation models (*n* = 336).

Predictor	*b*	SE	*t*	*p*	SE (HC3)	p (HC3)
Model 1, mediator equation (goal-oriented motivation)						
BID	0.455	0.052	8.740	< 0.001	0.061	< 0.001
ESE	0.099	0.060	1.646	0.101	0.073	0.177
BID × ESE	0.465	0.056	8.299	< 0.001	0.081	< 0.001
*R*^2^ = 0.286, *F*(3, 332) = 44.34, *p* < 0.001						
Model 1, outcome equation (compensatory exercise behavior)						
BID	0.269	0.058	4.640	< 0.001	0.071	< 0.001
GM	0.328	0.055	5.947	< 0.001	0.078	< 0.001
ESE	0.076	0.061	1.251	0.212	0.066	0.249
BID × ESE	0.323	0.062	5.221	< 0.001	0.090	< 0.001
*R*^2^ = 0.326, *F*(4, 331) = 39.94, *p* < 0.001						
Model 2, mediator equation (shame)						
BID	0.259	0.058	4.491	< 0.001	0.061	< 0.001
ESE	−0.094	0.067	−1.408	0.160	0.064	0.142
BID × ESE	−0.170	0.062	−2.740	0.007	0.060	0.005
*R*^2^ = 0.091, *F*(3, 332) = 11.06, *p* < 0.001						
Model 2, outcome equation (exercise avoidance behavior)						
BID	0.216	0.052	4.173	< 0.001	0.057	< 0.001
Shame	0.427	0.048	8.914	< 0.001	0.056	< 0.001
ESE	−0.058	0.058	−0.994	0.321	0.056	0.299
BID × ESE	−0.138	0.055	−2.530	0.012	0.054	0.011
*R*^2^ = 0.307, *F*(4, 331) = 36.67, *p* < 0.001						

BID, body image disturbance; ESE, exercise self-efficacy; GM, goal-oriented motivation; CEB, compensatory exercise behavior; EAB, exercise avoidance behavior. Coefficients are unstandardised (*b*). Body image disturbance and exercise self-efficacy were mean-centered before the interaction term was formed. SE and *p* are ordinary least squares values; SE (HC3) and *p* (HC3) are the corresponding HC3 heteroscedasticity-consistent values. Exact *p* values are reported and no significance asterisks are used.

White's general test was significant in all four equations, LM = 111.11, df = 8, *p* < 0.001, and LM = 68.92, df = 13, *p* < 0.001, in the first model and LM = 21.09, df = 8, *p* = 0.007, and LM = 24.62, df = 13, *p* = 0.026, in the second; the Koenker studentized Breusch-Pagan test was significant only in the two equations of the second model. Under HC3 standard errors, reported in Table 8, every interaction term remained significant in the same direction. No case exceeded a Cook's distance of 1, the largest being 0.336, and in the leave-one-out analysis the index of moderated mediation ranged from 0.144 to 0.162 and from −0.080 to −0.061, so neither result depended on a single observation.

## 5 Discussion

### 5.1 The differential associations of body image disturbance with compensatory and avoidance exercise behaviors

The findings of this study indicate that body image disturbance is positively associated with both compensatory exercise behavior and exercise avoidance behavior, which suggests that students who report body image disturbance may either report more exercise directed at self-improvement or report avoiding exercise because of negative emotions. This finding reaffirms the double-edged role of body image disturbance in exercise behavior and goes some way toward reconciling the inconsistent directions reported in existing research.

The positive association with compensatory exercise behavior aligns with studies grounded in self-regulation theory (Latinjak, 2025). Such research generally holds that when individuals perceive a gap between their actual physique and an ideal standard, compensatory motivation arises and the gap is addressed through means such as exercise (Buchholz and Crowther, 2014; Tomczak et al., 2021). The association observed here is therefore consistent with a goal-oriented regulatory process organized around perceived self-discrepancies (Briki, 2020).

The present study also found that body image disturbance was positively associated with exercise avoidance behavior, which is not entirely consistent with previous studies suggesting that body image disturbance either accompanies more exercise or is unrelated to it (Glashouwer et al., 2019; More et al., 2019; Tornero-Quiñones et al., 2019). One reason for the discrepancy is that many studies leave the emotional pathway out of consideration (Vocks et al., 2009). Drawing on self-conscious emotion theory, the present study indicates that body image disturbance does not consist solely of an appraised discrepancy, since the preoccupation and interference it entails are also associated with self-conscious emotions such as shame (Ahorsu et al., 2023; Raspovic et al., 2023), and hence with avoidance in exercise contexts involving bodily exposure or social comparison. The role of body image disturbance is thus not confined to a single pathway but reflects two pathways operating together, one motivational and one emotional (Messer et al., 2025).

### 5.2 The mediating roles of goal-oriented motivation and shame

#### 5.2.1 The mediating role of goal-oriented motivation

Goal-oriented motivation accounted for part of the association between body image disturbance and compensatory exercise behavior. This suggests that body image disturbance is not directly reflected in behavior but is accompanied by a motivational process (Salvador et al., 2023). The finding is consistent with achievement goal theory and with findings in exercise psychology (Hogue, 2020), according to which individuals who regard their physical condition as a target for improvement acquire a clear direction of effort and engage more actively in exercise (Czepczor-Bernat, 2022).

The present findings indicate that body image disturbance may be associated with different exercise responses depending on the accompanying cognitive and emotional processes. However, the compensatory response should not be interpreted as necessarily beneficial or adaptive (Salvador et al., 2023). This discrepancy may stem from the neglect of individual cognitive appraisals in previous research. According to the biopsychosocial model of challenge and threat validated by Blascovich et al. (1999), individuals with robust self-regulatory capacities perceive adequate internal coping resources to handle situational demands, thereby tending to appraise negative stimuli as challenges instead of threats.

#### 5.2.2 The mediating role of shame

Shame accounted for part of the association between body image disturbance and exercise avoidance behavior (Ryan et al., 2025). This finding is consistent with self-conscious emotion theory, which holds that shame, as an emotion oriented toward global self-denial (Huellemann et al., 2021), is accompanied by avoidance that serves to prevent further negative evaluation.

The finding is also consistent with previous research reporting that shame about the body accompanies lower physical activity participation (Huellemann et al., 2024). The present study further indicates that this negative association is not direct but is statistically accounted for by the emotional experience associated with body image disturbance (Smith et al., 2024).

Variation in this pathway across studies may be attributable to situational differences such as the presence or absence of evaluation by others, to individual differences such as sensitivity to shame, and to differences in measurement (Tang and Shao, 2026).

### 5.3 The moderating role of exercise self-efficacy

First, in the emotional pathway linking body image disturbance to shame, the moderation was significant and negative (Ouyang et al., 2020; Wang et al., 2024). Students with high exercise self-efficacy may regard physical discrepancies as controllable and open to change through effort, which is associated with less self-denial and with weaker shame, whereas those with low self-efficacy are more likely to interpret the perceived discrepancy as a stable personal flaw, which is associated with stronger shame (Niu et al., 2025; Wang et al., 2024). This is consistent with a cognitive appraisal account, and with the view in social cognitive theory that self-efficacy shapes how threatening information is interpreted (Charokopaki and Douros, 2025; Lucibello et al., 2025).

Second, in the motivational pathway linking body image disturbance to goal-oriented motivation, the moderation was significant and positive (Chu et al., 2021; Sheng et al., 2025). Students with high self-efficacy may be more likely to interpret body image disturbance as a goal that can be attained through their own effort, which is associated with a self-improvement goal structure and with clearer behavioral plans (Zhao et al., 2023). Self-efficacy in this pathway is associated not only with expectations of goal attainment but also with persistence when difficulties arise (Strachan and Brawley, 2008). For students with low self-efficacy, by contrast, body image disturbance is more often accompanied by a sense of helplessness about the possibility of change, and goal-oriented motivation is correspondingly weaker (Lynn et al., 2022). The role of self-efficacy in this pathway therefore appears to lie in the perceived feasibility of action.

Exercise self-efficacy was associated with the strength of both mediating pathways at once (Honicke et al., 2020; Strachan and Brawley, 2008). At high levels, the indirect association through goal-oriented motivation was stronger and that through shame was no longer significant, whereas at low levels the emotional pathway was relatively more prominent (An et al., 2024; Meixner et al., 2025). Self-efficacy therefore appears to alter the relative weight of the two pathways rather than to operate through one of them alone.

This pattern offers one account of the inconsistent directions reported in previous studies. Differing conclusions about whether body image disturbance accompanies more or less exercise may reflect variation in participants' levels of self-efficacy, since the motivational pathway is more prominent in samples with generally high self-efficacy and the emotional pathway in samples with lower self-efficacy (An et al., 2024; Meixner et al., 2025). The present study therefore adds a more differentiated account of the complexity of the association between body image disturbance and exercise behavior.

### 5.4 Alternative directional and reciprocal interpretations

Because all variables were measured at a single time point, the observed pattern of associations is compatible with several directional accounts. The reverse direction is plausible, in that appearance-oriented exercise goals and compensatory exercise may be accompanied by greater body surveillance and hence by heightened body image disturbance rather than following from it (Burgon and Waller, 2024).

Reciprocal accounts are equally plausible. Body image disturbance may be accompanied by compensatory exercise, which in turn sustains appearance focus and maintains the disturbance, whereas exercise avoidance may reduce feedback about physical competence and maintain the disturbance in a different way (Guimond et al., 2026).

The mediators and the moderator may themselves be shaped by repeated behavioral experience. Shame in exercise settings and exercise self-efficacy can both develop through an accumulation of exercise experiences, so within the present design they can be regarded as outcomes of exercise behavior as readily as antecedents of it.

### 5.5 Limitations and future directions

Several limitations of this study should be acknowledged. They also suggest directions for future research.

First, the design limits the scope of the inferences that can be drawn. The cross-sectional data preclude causal inference and cannot establish temporal ordering among the variables. The convenience sample of Chinese university students further limits generalizability, since appearance norms and social evaluative pressures may differ in other cultural settings and populations. Longitudinal and experimental designs would help to establish temporal precedence and replication in other cultural settings, in non-student adults and in individuals with clinically significant body image disturbance would strengthen external validity.

Second, all focal constructs were measured using similarly formatted self-report scales administered to the same respondents at the same time. Neither the single-factor diagnostic nor the method-factor model can rule out same-source measurement bias, and the estimates should be read with this in mind. Future studies should use marker variables, temporal separation, multi-source assessment or objective measures of exercise.

Third, confounder control was limited to gender and age, because data on other plausible common causes such as body mass index, exercise and sport participation, eating or weight-control behaviors, mental health symptoms and social evaluative concerns were not collected. Their absence cannot be remedied statistically, and residual confounding may affect the estimates.

Fourth, the measures were translated and adapted for this study and their psychometric evidence derives from the present sample, so independent validation would be desirable; in particular, the compensatory exercise measure captures the compensatory function of exercise without distinguishing adaptive from compulsive forms, and the pathway through goal-oriented motivation should not be read as evidence of healthy self-regulation.

## 6 Conclusion

Drawing on self-regulation theory, self-conscious emotion theory and social cognitive theory, this study specified and tested a dual-path moderated mediation model of the associations between body image disturbance and exercise behavior. Body image disturbance was positively associated with both compensatory exercise behavior and exercise avoidance behavior, reflecting two contrasting patterns of exercise response. Goal-oriented motivation partially accounted for the association between body image disturbance and compensatory exercise behavior, and shame partially accounted for the association between body image disturbance and exercise avoidance behavior. Exercise self-efficacy was associated with a stronger motivational pathway and a weaker emotional pathway. Because these patterns are cross-sectional, exercise self-efficacy is best regarded as a promising candidate target for future longitudinal and intervention research rather than as an established lever for behavior change. The contribution of the study lies in the empirical integration of two processes that have usually been examined separately, rather than in the identification of a new psychological mechanism, and the findings generate hypotheses to be tested with stronger designs before intervention recommendations are drawn.

## Data Availability

The raw data supporting the conclusions of this article will be made available by the authors, without undue reservation.
