# Supplementary material for: Body image disturbance, compensatory exercise, and exercise avoidance: cross-sectional conditional process associations in university students
Source: Front Psychol. 2026 Aug 31;17:1917371. doi: 10.3389/fpsyg.2026.1917371 (PMC13572585; doi:10.3389/fpsyg.2026.1917371)
Supplement: Supplementary file 1 [file Supplementary_File_1.docx]

Appendix A

Table A1. Complete list of scale items and their sources.

| **Construct** | | | **Items** | **References** |
| --- | --- | --- | --- | --- |
| Body image disturbance (BID) | | | | |
| BID1 | I pay a lot of attention to my body image. | | | (Cash et al., 2004) |
| BID2 | I often imagine my body's "flaws". | | |  |
| BID3 | My concern about my body shape makes me feel anxious or troubled. | | |  |
| BID4 | This anxiety about my body shape can affect my studies, work, or daily life. | | |  |
| BID5 | I avoid social or group activities because I'm worried about my figure. | | |  |
| BID6 | My body image disturbance sometimes affects my education, career, or daily performance. | | |  |
| Exercise self-efficacy (ESE) | | | | |
| ESE1 | Even with a heavy workload, I manage to maintain a regular weekly physical activity. | | | (Chen et al., 2019; Motl et al., 2000) |
| ESE2 | Even without anyone by my side, I can complete physical activities independently. | | |  |
| ESE3 | Even with temptations such as TV or games, I still make sure to exercise during most of my free time. | | |  |
| ESE4 | Even when the weather is bad, I can still do physical activities as planned. | | |  |
| ESE5 | I can get my friends or classmates to exercise with me most of the time. | | |  |
| ESE6 | I can complete physical activities even in my dormitory or at home. | | |  |
| ESE7 | I can proactively schedule my workouts because I know how to do it. | | |  |
| ESE8 | No matter how busy I am, I can stick to my exercise plan most of the time. | | |  |
| Shame | | | | |
| Shame1 | When I don’t perform well in sports or workouts, I feel like a failure or unacceptable. | | | (Hoppen et al., 2022) |
| Shame2 | I worry that others will look down on or despise me because of my athletic ability or physical performance. | | |  |
| Shame3 | I feel ashamed when I do something embarrassing during sports or exercise. | | |  |
| Shame4 | I feel ashamed when I think about the possibility that others might have negative comments about my athletic performance or physical appearance. | | |  |
| Goal-oriented motivation (GM) | | | | |
| GM1 | I feel successful when I learn new skills in sports. | | | (Duda & Nicholls, 1992) |
| GM2 | When I feel I've made progress, I feel like I've done well. | | |  |
| GM3 | I feel satisfied when I try new moves. | | |  |
| GM4 | I feel successful when I do better than before. | | |  |
| GM5 | I felt I performed well when I understood how to improve my athletic skills. | | |  |
| Compensatory exercise behaviour (CEB) | | | | |
| CEB1 | | When I eat more, I compensate by exercising extra. | | (Knäuper et al., 2004; Yuan et al., 2024) |
| CEB2 | | After exercising, I feel like I can relax my dietary restrictions. | |  |
| CEB3 | | If I don’t exercise on a certain day, I will increase the amount of exercise in the following days. | |  |
| CEB4 | | I often exercise extra because I'm worried about my weight or appearance. | |  |
| CEB5 | | For me, exercise is sometimes a way to make up for not exercising or overeating. | |  |
| Exercise avoidance behaviour (EAB) | | | | |
| EAB1 | | I try to avoid exercising when it makes me feel embarrassed or uncomfortable. | | (Gámez et al., 2011) |
| EAB2 | | I avoid participating in sports activities for fear of performing poorly. | |  |
| EAB3 | | When I’m stressed or in a bad mood, I tend to avoid exercising. | |  |
| EAB4 | | I will avoid exercising to avoid possible failure or being evaluated. | |  |
| EAB5 | | Sometimes I choose not to exercise because of the fatigue or pain that exercise may cause. | |  |
